# Sex, Gender, and Alcohol Use: Implications for Women and Low-Risk Drinking Guidelines

**DOI:** 10.3390/ijerph19084523

**Published:** 2022-04-08

**Authors:** Lorraine Greaves, Nancy Poole, Andreea C. Brabete

**Affiliations:** 1 Centre of Excellence for Women’s Health, Vancouver, BC V6H 3N1, Canada; npoole@cw.bc.ca; 2 School of Population and Public Health, Faculty of Medicine, University of British Columbia, Vancouver, BC V6T 1Z3, Canada

**Keywords:** alcohol, drinking guidelines, sex-related factors, gender-related factors, health promotion, health policy, sex and gender science, women’s health

## Abstract

Alcohol use is coming under increasing scrutiny with respect to its health impacts on the body. In this vein, several high-income countries have issued low-risk drinking guidelines in the past decade, aiming to educate the public on safer levels of alcohol use. Research on the sex-specific health effects of alcohol has indicated higher damage with lower amounts of alcohol for females as well as overall sex differences in the pharmacokinetics of alcohol in male and female bodies. Research on gender-related factors, while culturally dependent, indicates increased susceptibility to sexual assault and intimate partner violence as well as more negative gender norms and stereotypes about alcohol use for women. Sex- and gender-specific guidelines have been issued in some countries, suggesting lower amounts of alcohol consumption for women than men; however, in other countries, sex- and gender-blind advice has been issued. This article reports on a synthesis of the evidence on both sex- and gender-related factors affecting safer levels of drinking alcohol with an emphasis on women’s use. We conclude that supporting and expanding the development of sex- and gender-specific low-risk drinking guidelines offers more nuanced and educative information to clinicians and consumers and will particularly benefit women and girls.

## 1. Introduction

While many high-income countries have issued advice suggesting pregnant women should not drink alcohol [1], few have addressed more general sex- and gender-related factors in forming health advice about alcohol use. Canada, for example, issued sex-specific guidance in 2012 [2], and the USA embedded such advice within its dietary guidelines in 2015 and 2020 [3,4]. In the same time period, the guidance regarding safe consumption of alcohol issued in Australia [5], the UK [6], France, and Netherlands [7,8] was sex and gender neutral, resulting in one set of advice and guidance for all people.

Canada’s 2012 Lower Risk Drinking Guidelines recommend that women consume less alcohol than men in per day, week or at a sitting, in order to safeguard their health. In the USA in 2015 and 2020, sex-specific advice was released, recommending that women drink one drink or less per day and no more than three in one sitting; less than the two drinks and five drinks suggested for men [9,10]. In numerous other countries, such as New Zealand, Italy, Spain and Germany, sex-specific guidance has been issued, albeit with widely varying suggested weekly levels of safe consumption, over the course of the same decade [7,8]. Hence, in relatively comparable countries, assessment of the evidence on the differential impacts of alcohol on males and females and men and women has resulted in different advice and guidance. 

This anomaly may reflect the nation-based capacity in, or commitment to sex and gender science, policies on sex and gender-based analysis, interpretations of scientific evidence, cultural attitudes regarding alcohol, commitments to women’s health, system structures for issuing advice, or public demands for such information. In any case, there is evidence suggesting that sex- and gender-related factors affect alcohol use and its impacts with particular risks for women that could directly impact the design and calculation of lower risk drinking guidelines. This article reviews this material with a view to interpreting sex-based (biological) information about the effects of alcohol on female or male bodies as well as gender-based (sociocultural) information that indicates differential effects or impacts on men, women, and gender diverse individuals.

## 2. Background

Sex- and gender-related factors affect the use of all substances, including alcohol. The concepts of sex and gender include many components relevant to health [11], and yet have been consistently under researched [12]. Nevertheless, sex differences in the impact of alcohol have been identified for at least four decades [13], indicating more negative impacts on female bodies, ranging from faster intoxication [14,15], more disease related damage on lower amounts of alcohol [15,16], and shorter routes to dependence in treatment populations [17]. Sex is often used simply as a comparator and category in population research or as an observed characteristic in experimental research or clinical practice. However, it is vital to understand that in fact a range of *sex-related factors* affect health that do not rely on categorizing or comparisons between the sexes (differences) but require research, analysis, and consideration in their own right. 

With respect to women’s health and alcohol use, it is important to clarify which aspect of sex may be of importance in developing and denoting specific advice regarding alcohol consumption. For example, evidence shows that pharmacokinetics and pharmacodynamics affect the processing of alcohol including the absorption, distribution, metabolism, and elimination of drugs. Mechanisms such as drug clearance are linked to sex-related factors in the expression of metabolic enzymes [18,19] and renal clearance of drugs is decreased in females because of a lower glomerular filtration rate compared to males [20]. 

A systematic review on sex differences in pharmacotherapy showed that females generally have lower gastric emptying times, gastric pH, lean body mass, and hepatic clearance but higher plasma volume, BMI, and body fat, which, when coupled with a difference in Cytochrome P450 enzyme activity, can all contribute to a difference in the rate of drug metabolism compared with males [21]. These factors affect the break down and absorption of alcohol resulting in higher blood alcohol concentrations and greater volume of distribution, compared to males. In general, alcohol is processed differently in female and male bodies, which is similar to other drugs [22]. 

Gender-related differences and factors are also at play in influencing the real-life experiences of all who drink alcohol as gendered norms and stereotypes impact the behaviour of all people. With respect to alcohol, women, men and gender and sexual minority group members experience different social expectations, pressures, and responses. For example, women and girls are often deterred by social mandates from consuming alcohol or becoming intoxicated [23], while men and boys, in some settings, are implicitly encouraged via prevailing expressions of masculinities [24]. 

Girls and women who are pregnant or mothering experience additional stigmatizing attitudes toward drinking alcohol and are judged accordingly, in some cases with state-sanctioned interventions, while partners and fathers are exempt from such reprobation. In addition, vulnerabilities to violence or assault are differentially distributed, with girls and women vulnerable to sexual assault [25] and intimate partner violence (IPV) and men and boys to violence and aggression as a result of drinking excessively or intoxication [26]. All of these factors are at play in assessing the risk, safety, and impacts of alcohol use particularly on women and girls. 

## 3. Methods

We conducted a comprehensive synthesis of the recent literature focused on identifying recent evidence on alcohol risk factors and related health outcomes. Topic areas and the parameters for risk factors were initially informed by (a) the alcohol section of a 2018 scoping review conducted on sex, gender, and four substances: alcohol, cannabis, nicotine/tobacco, and opioids, described elsewhere [11]. We then conducted an additional search designed by a library and information specialist between July and October 2021 that is described in the next section.

### 3.1. Search Strategy 

The 2021 literature search was conducted by an information specialist at the Canadian Centre on Substance Use and Addiction (CCSA) using health-related databases with international coverage (Medline, Embase, Cochrane Database of Systematic Reviews, and Cochrane Central Register of Controlled Trials via Ovid; CINAHL, PsycINFO, Social Work Abstracts, Women’s Studies International, and LGBT Life via EbscoHost; and Social Science Citation Index via Clarivate Analytics) for English language articles. This search identified research published between 2018 and 2021 that addressed sex and/or gender-related factors and alcohol use. The search terms used for this search can be found in Table S1.

### 3.2. Inclusion/Exclusion Criteria

Specific inclusion and exclusion criteria were developed for this search. In general, the search included English language, peer-reviewed journal articles that included systematic reviews and meta-analyses and individual studies that contained information on the risk factors and health outcomes associated with alcohol use. We primarily focussed on recent systematic reviews and meta-analyses. Subject areas where systematic review evidence was limited (such as women’s health during pregnancy) were supplemented by reviewing individual studies. 

In alignment with a previous 2018 scoping review [11], we included literature published between 2007 and 2021 from the following high-income countries: Australia, Austria, Belgium, Canada, Denmark, Finland, France, Germany, Greece, Iceland, Ireland, Italy, Luxembourg, Netherlands, New Zealand, Norway, Portugal, Spain, Sweden, Switzerland, United Kingdom, and the United States. Studies published in all other countries, or that include data from multiple countries where the data were not disaggregated, were excluded. 

### 3.3. Analysis and Synthesis

All authors met weekly to discuss the findings, including alcohol-related risk factors and related health outcomes. A narrative summary approach was used to succinctly synthesize the main findings and implications of the identified literature. 

## 4. Results

### 4.1. Sex-Related Factors

There are several types of sex-related impacts of alcohol that affect male and female bodies differently including genetic, anatomical, and physiological structures and processes. These aspects affect the impact of alcohol on organs, disease processes, and conditions, as well as the immediate impacts of alcohol use on intoxication and impairment. 

#### 4.1.1. Metabolism

In many cases, female and male bodies respond differently to alcohol as a result of different pharmacokinetic (PK) processes. In general, women have a lower volume of body water and a higher volume of body fat than men of similar body weight. The first-pass metabolism of ethanol is greater in males than in females and the volume of distribution is less in females compared to males [27]. These PK differences might explain why women show greater blood concentrations of alcohol [27]. Importantly, females break down ethanol faster than males [28] and reach a higher blood alcohol concentration due to faster absorption [29]. Hormones might also play an important role as increased serum progesterone levels are associated with faster alcohol elimination rates in women but not in men [30]. 

#### 4.1.2. Impact on Disease Processes

Alcohol also affects a range of disease processes, organs, systems, and conditions, and there are sex-based differences in the pathophysiological consequences of consuming alcohol. For example, a study that examined cardiac reactivity measures after acute alcohol ingestion revealed that the heart rate variability measure, pNN50, reflected significant interactions between alcohol and sex with females exhibiting higher values than males [31]. Further, even low-to-moderate alcohol consumption is associated with an increased risk of cancers of the oral cavity and pharynx, oesophagus, larynx, rectum, liver, breast, and all cancers combined in a study conducted among women [32]. Indeed, the consumption of alcohol is causally linked to cancer of the female breast, with factors such as nutrition, hormonal state, menopause, adolescent drinking and genetic and epigenetic susceptibilities possibly playing a role [33].

Sex-related factors play an important role in the development of alcoholic liver cirrhosis and in the progression of alcoholic liver disease [34]. In a study conducted with male and female alcohol-dependent subjects admitted to an alcohol treatment program, females showed higher levels of biomarkers of liver injury than males even though they used less alcohol on a daily basis and had been drinking for shorter time periods [35]. These findings suggest that females develop and have more progressive liver injuries, even when consuming lower quantities of alcohol [36]. Indeed, in a systematic review and meta-analysis on the impact of alcohol as a risk factor for liver cirrhosis, women were more impacted than men by the same amount of drinking in both mortality and morbidity studies [37].

#### 4.1.3. Hormones

Hormonal changes during pregnancy, menopause, or after ingesting oral contraception have an impact on PK processes by affecting the performance of enzymes, metabolism, and clearance processes for women [38]. Alcohol may also act to alter sex hormone levels. For example, alcohol infusions provoked differential responses in women and men when administered in a two-session single-blinded study. While the level of testosterone decreased in men, the level of estradiol increased in women [39]. 

In another study with pre-menopausal women, evidence suggested that the previous day’s alcohol intake, notably of wine and beer, was significantly associated with elevated total and free estradiol, testosterone, and luteinizing hormone (LH), and binge drinking magnified these effects [40]. Hormones may also affect the development of Alcohol Use Disorder (AUD). In a systematic review of 50 articles (19 conducted with humans and 31 with animals) on the impacts of sex hormones on alcohol consumption and AUD, Erol et al., (2019) found evidence of an association of increased testosterone level and increased risk for alcohol use and AUD in males [41]. Among females, there is support for a positive relationship between increased oestrogen level and increased alcohol use with mixed results found in males [41]. 

#### 4.1.4. Genetics

The genetic influences on AUD may also be sex-specific. In a Swedish study with national data from 787,916 twin and sibling pairs, Kendler et al. (2016) found that there are sex differences in the aetiology of AUD with weaker genetic effects among females than among males [42]. In another study, males showed a stronger externalizing pathway to genetic risk to AUD than females [43].

#### 4.1.5. Impact on the Brain

There are numerous indications that brain function post-alcohol use is different in males and females. Experimental studies measured the neurobiological impacts of various levels of administration of alcohol in males and females. For example, Rickenbacher et al. (2011) [44] investigated the effects of acute alcohol intoxication on grey matter perfusion in males and females using arterial spin labelling (ASL) to specifically examine regional brain impacts. Acute intoxication increased perfusion in bilateral frontal regions in males but not in females. Under placebo, stronger cortical perfusion was observed in the female subjects when compared to the males, primarily in the left hemisphere in frontal, parietal, and temporal areas. These results emphasize both sex-related impacts and differences in regional specificity of alcohol’s effects of cerebral perfusion possibly because of interactive influences on hormonal, metabolic, and hemodynamic autoregulatory systems. Alcohol-induced perfusion increases correlated positively with impulsivity/antisocial tendencies, which is consistent with dopaminergic mediation of reward and its effects on cortical perfusion.

Even at lower dosages, experiments revealed impacts on inhibition. For example, Hoppenbrouwers et al. (2010) [45] examined sex differences in frontal interhemispheric connectivity in response to alcohol, with 12 female and 10 male healthy volunteers who received a single administration of 0.5% alcohol in a placebo-controlled counterbalanced crossover design. Paired-pulse transcranial magnetic stimulation was applied to measure transcallosal inhibition (TCI) between the left and right primary motor cortex (M1). The administration of a single oral dose of alcohol resulting in a blood alcohol concentration of 0.05% reduced transcallosal inhibition between the right and left primary motor cortex in females but not males. 

Sex-related factors and differences in the neurobiological impacts of alcohol use also have potential implications for behavioural patterns between men/boys and women/girls. A recent narrative review of neuroimaging findings in alcohol use over the last 10 years found that, overall, adolescent girls demonstrated smaller prefrontal cortex volumes and less frontal activation compared to same-sex controls. Among boys the opposite was found, suggesting that these findings may have implications for sex-related cognitive deficits associated with alcohol use [46]. 

For example, in a study on the associations between binge drinking and the spatial working memory brain activation, males with adolescent-onset AUD or binge-drinking males had greater frontal activation in response to a spatial working memory task compared to same-sex controls. Females with adolescent-onset AUD or binge-drinking females had less frontal activation to a spatial working memory task compared to same-sex controls [47]. For female binge drinkers, less frontal activation was associated with poorer working memory and attention on the spatial working memory task [47]. 

Sex-related differences and factors may also impact both subjective and objective experiences of alcohol ingestion and may manifest differently in those with AUD compared to binge or social drinkers. For example, a study on alcohol cue-induced activation showed that women with AUD activated different reward circuits, cognitive control circuits, and regions of the default-mode network compared to same sex controls [48]. The authors suggested that these results might indicate a problem with switching between different neural networks. The impacts of binge drinking are also sex-specific as acute intoxication increased cortical perfusion in bilateral frontal regions in men but not in women [44]. 

Another study conducted with healthy social drinkers in which they participated in both alcohol (0.60 g/kg ethanol for men and 0.55 g/kg for women) and placebo conditions found that women reported feeling more intoxicated than men and had lower activity in their anterior cingulate cortex than men in a modified four-colour Stroop task that combined reading and colour naming and used manual responses. These findings show that when alcohol interferes with goal-directed behaviour resulting in poor self-control, women might be affected more than men [49].

It is possible that neurobiological sex differences persist post recovery from AUD, influencing ongoing brain health. Evidence from a study that examined drinking history associations with regional white matter volumes in abstinent alcoholic men and women reported differences by sex. Women were more impacted in the frontal, temporal, ventricular, and corpus callosum regions, while men showed effects mainly in the corpus callosum [50]. 

Finally, alcohol may have sex-differential impacts on impairment, measured both subjectively and objectively. Administration of a single oral dose of alcohol resulting in a blood alcohol concentration of 0.05%, reduced transcallosal inhibition between the right and left primary motor cortex in women but not in men [45]. An experiment by Miller et al. (2009) [51] revealed that men and women respond differently to a dose of alcohol calculated by body weight (0.65 g/kg). Although alcohol significantly impaired all aspects of driving performance in which cognitive functions, such as motor coordination, speed of information processing, and information-processing capacity, are involved, women displayed greater impairment than men on these behavioural tests and reported higher levels of subjective intoxication compared with men [51].

#### 4.1.6. Telescoping

Women have shorter intervals between the initiation of alcohol use to entering treatment, experience medical and health-related problems earlier even when they consume the same amount of alcohol as men and have different severity of cognitive consequences reflecting both sex- and gender-related factors, such as both emotional and social factors [52]. This escalation of use, sometimes referred to as the telescoping effect, is more pronounced in treatment samples than population-based samples [53]. For those in treatment, the length of time from first alcohol use to the onset of alcohol-related problems and their consequences is faster among women than men [52]. 

There are sex-related factors associated with the telescoping effect, such as the rate at which alcohol is metabolized, a greater sensitivity to alcohol in women [54], and life-stage factors that determine use of hormonal contraception or hormone levels, that also interact with gendered and social factors. For example, women with a history of childhood maltreatment are particularly vulnerable to an accelerated time from initiation of alcohol use until dependence [55]. Taken together, the causes and effects of sex-specific differences and influences linked to alcohol use (more precisely termed ‘sex-related factors’) are complex and interact in influential ways with gender-related factors on women’s health.

### 4.2. Gender-Related Factors

**Figure 1:**
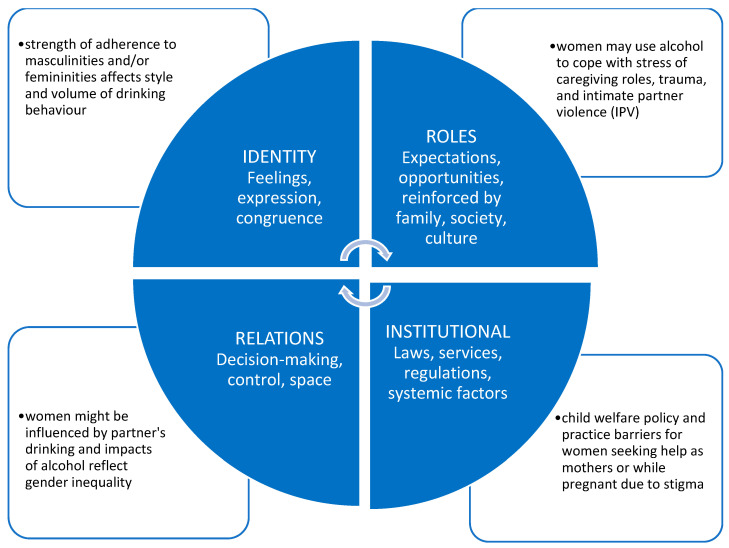
Gender-Related Factors. Adapted from Greaves L. and Hemsing N. 2020 [56].

The impact of gender on alcohol use includes the influences of roles, relations, identities, and institutional practices, which work together to make up gender. These elements are temporal and culturally specific and interact with sex-related factors to produce effects or impacts, and potentially have differential impacts in different countries or societies. Figure 1 presents the main categories of the gender concept, with an example of the impact of alcohol in each quadrant.

Our search for systematic reviews published since 2017 related to gendered influences on women’s alcohol use identified 18 results, ten of which focussed on the gender influences on pregnant women and mothers. 

#### 4.2.1. Gender Norms

The prevailing social roles are the standard or normative behaviours expected of girls, boys, women, and men, including norms and behaviours regarding the use of, amount of, or intoxication related to alcohol use in a particular cultural or subgroup context. Roles and expectations regarding masculinities and femininities are well-established in most cultures and are reinforced through strong socialization processes and the media. Gender-diverse individuals may seek to conform to the norms associated with a gender incongruent with their sex, or non-binary individuals may reject all gendered norms. 

The prevailing norms, for example, may excuse intoxication among boys more than among girls. Stigma or negative attitudes about alcohol use may be applied to women more often than men and mothers more often than fathers, as another example. Further, these norms may shift over time as, for example, the tolerance for binge drinking among girls has increased, while the social tolerance for alcohol use by pregnant women has decreased in recent decades.

Two systematic reviews focussed on gender norms and roles. One review examined the evidence between conformity with gender norms and alcohol use and/or abuse in adults [57]. Conformity to norms associated with traditional masculine roles (dominance, ‘womanizing’, aggressiveness, and risk behaviours) is related to greater alcohol use) conformity to norms associated with traditional feminine roles (interest in home life and family care) is related to lower alcohol use. These findings provide insights into the relationship between gender and drinking. 

Another review found norms related to mothering as influential in women’s health behaviour during pregnancy, specifically related to dietary behaviour, physical activity, smoking, and alcohol use [58]. Three overarching themes were noted (1) time to think about ‘me’, (2) adopting the ‘good mother’ role, and (3) beyond mother and baby. These findings provide an improved understanding of the various dynamic changes in internal and external factors influencing women’s health behaviour during the antenatal period. The possibilities of modifying gendered beliefs and patterns linked to risk behaviours is an important aspect of prevention, and more precise development of gender measures is necessary to further deepen the study of these relationships.

#### 4.2.2. Gender Relations

Relationships between people in romantic, sexual, household, work, or friendship settings are typically gendered. In heterosexual relationships, there is often an imbalance in decision making power on issues such as freedom of movement or behaviour, including alcohol and drug use, spending money, driving vehicles, or recreational pursuits. Members of any kind of couple relationship can, and do, influence each other to drink, drink more, drink less, or not at all, with or without coercion. 

These couple dynamics have been shown to be important with respect to substance use patterns, and often mix with other factors or dynamics, such as intimate partner violence (IPV), pregnancy and parenting, or addiction. Several of the recent reviews focussed on the influence of IPV and coercive relationships. This long-standing gendered influence on women’s alcohol use points to a need for tailored trauma and gender-informed approaches in messaging about drinking guidelines. 

One systematic review examined research in this area from 2012 through 2019 [59]. Another systematic review described what is known about the prevalence, risk factors, and health consequences associated with IPV among young pregnant women [60]. Risk factors associated with IPV during pregnancy included having a husband/partner with a low education level, a low level of family income, and partners’ problem drinking. Protective factors included sex education for girls, youth services, and reducing gender inequality. Without promoting gender equality, the problem of IPV is likely to continue. More culturally tailored intervention research addressing this among various populations is needed. 

Gendered relations can be directly invoked in prevention efforts. For example, encouraging reciprocal partner support to reduce vulnerability to perinatal depression and anxiety can include actions on becoming a parent, supporting each other through pregnancy and childbirth, communication, conflict, division of labour, practical support, emotional support, emotional closeness, sexual satisfaction, using alcohol and drugs, encouraging self-care, developing acceptance, and help-seeking [61]. 

Other gendered relational factors also matter in assessing risky alcohol use by women, such as vulnerability to sexual assault. One systematic review examined the evidence on the effects of alcohol intoxication on sexual assault risk information processing among young adult women [62]. Thirteen of the fourteen identified studies report at least partial support for intoxication impairing the attention to cues, interpretation of social information, or intended behavioural response in a hypothetical sexual assault scenario. 

Another systematic review identified some psychosocial correlates of sexually transmitted and blood borne infection (STBBI) acquisition, unplanned pregnancy, abortion, and risky sexual behaviours in general population samples of women of reproductive age [63]. Multiple partnerships were associated with intensity of marijuana and alcohol use, and smoking. These relational gender influences on women’s alcohol use prompt consideration of settings, such as schools, colleges, and sexual health clinics, as locations for sharing lower risk alcohol use guidelines. In short, gendered relations are not to be ignored in considering the content and impact of messaging surrounding drinking guidelines.

#### 4.2.3. Gender Identity and Sexual Orientation

In general, people who are in either sexual minority or gender minority groups consume more alcohol than those in majority groups. However, reliance on non-representative samples, and a range of other methodological limitations are drawbacks in this research [64,65]. The degree to which any individuals conform to or resist prevailing femininities and masculinities, express gender in conforming or non-conforming ways, or claim or identify with a particular overarching gender identity, such as woman, man, trans (masculine or feminine), or non-binary, are all factors in how alcohol use might play out for groups of people. In addition, different sexual orientation groups, such as heterosexual, homosexual (gay or lesbian), or bisexual, may subscribe to different dimensions of femininity or masculinity along with experiencing sexual attraction to same sex persons. 

No systematic reviews were found specifically related to alcohol and gender identity. However, several individual studies show comparable gender influences on drinking for young people identifying as transgender, including influences, such as the links between alcohol use and risky sexual behaviour and with identity formation as college students [66,67,68]. One study found that high life stress was associated with an increased odds of sexual risk for young transgender women, and that this was attenuated by alcohol and other substance use. They concluded that interventions aimed at reducing sexual risk behavior in this population should address problems with alcohol and other substance use “as well as more distal factors that impact risk, such homelessness, joblessness, and lack of access to medical care” [68]. Another study concluded that transgender compared with non-transgender first-year students engage in higher-risk drinking patterns and experience more alcohol-related blackouts (ARBs) and other negative alcohol-related consequences (ARCs) [66]. The authors note that male-to-female transgender students had higher levels of alcohol consumption and frequency of alcohol-related blackouts and consequences than female-to-male transgender students. 

#### 4.2.4. Gendered Institutional Impacts

Gendered applications of policies, laws, regulations, and cultural prohibitions have an impact on different gender groups, particularly women. For example: warning signs regarding drinking during pregnancy in bar washrooms; stigma related to alcohol use for women, and pregnant women and mothers in particular; criminalization of pregnant women who drink in some jurisdictions; cultural and religious norms prohibiting alcohol use in women or men or both; and age guidelines for purchase all have differential impacts on men and women, boys, and girls. These policies and practices are all gendered in their impact or intent. 

Stigma and trauma are gendered issues and important aspects to be considered in prevention, treatment, and knowledge mobilization. These are particularly resonant when considering reproduction. One systematic review of qualitative studies involving pregnant and recently postpartum women was undertaken to understand the barriers and facilitators that influence alcohol use in pregnancy [69]. Five themes impacting women’s alcohol use, abstention and reduction were identified: (1) social relationships and norms; (2) stigma; (3) trauma and other stressors; (4) alcohol information and messaging; and (5) access to trusted equitable care and essential resources. However, the impact of structural and systemic factors on prenatal alcohol use was largely absent in the included studies, instead focusing on individual choice [69]. This represents a missed opportunity. 

Similarly, the availability of preconception health knowledge, messages, interventions, and programming is a crucial gendered aspect of understanding alcohol use. One systematic review examined how preconception health knowledge has been measured in the existing literature and identified measurement gaps, biases, and logistical challenges [70]. The authors noted that preconception health knowledge tools focused on fertility, folic acid, and alcohol, with few questions pertaining to men’s health, mental health, or the interconception period. 

Another systematic review explored enablers and barriers to women’s preconception lifestyle behaviours using several pre-established models and frameworks [71]. The presence/absence of knowledge on healthy behaviours was the most commonly assessed enabler/barrier. Building opportunities for preconception interventions where alcohol guidelines can be routinely discussed and giving support related to the importance of and confidence for change are important [72,73]. However, in the current context of an overloaded health care system, online preconception interventions may be the most feasible.

### 4.3. Sex-Gender Interactions and Intersectional Factors

In real life, sex and gender interact to determine the ultimate impacts on alcohol use by women and the consequences for women. Sex and gender interactions are enhanced when understood in an intersectional context and considering characteristics such as race/ethnicity, socioeconomic status, age, and other drug use. For example, sex and gender interact to stigmatize pregnant women and mothers regarding alcohol (and other substance) use [69], especially visibly pregnant women. 

This stigma is baked into a gendered institutionalized response in the form of birth alerts, child custody, and apprehension and welfare decisions that are state-specific and often punitive [74]. Such experiences can create long-term impacts on bonding, attachment processes, child development, and maternal and child mental health as well as contribute to ongoing trauma. Other gendered impacts include marketing, advertising, posters and warnings in bars, public places, and washroom walls along with depictions of alcohol use in media. 

Intimate partner violence (IPV) and alcohol use are also connected, illustrating another sex/gender interaction, as most victims of IPV are women. One systematic review described the negative effects of IPV on physical health outcomes for women, including worsening the symptoms of menopause, increasing the risk of diabetes, developing chronic diseases and pain, contracting sexually transmitted infections, and engaging in risk-taking behaviours, including the abuse of drugs and alcohol [59]. 

IPV also affects human immunodeficiency virus outcomes, worsening CD4+ cell depletion. This review highlighted significant gaps in this field of research in relation to cardiovascular disease, endocrine dysfunction, and neurological symptoms and conditions and underlines the need for additional long-term studies to better inform the health care of women who have experienced IPV and to establish the physiological mediators of these outcomes [59]. How alcohol may or may not be contributing to this range of health outcomes, as well as the association of alcohol use problems with IPV requires further study. 

Various additional factors and characteristics intersect with sex and gender to create outcomes for women [75]. There are complex interconnections that impact real life experiences for women who use alcohol or who feel the impact of others’ use of alcohol. One systematic review assessed the societal distribution of alcohol-attributable harm by investigating socioeconomic inequality and the related gender differences in alcohol-attributable mortality [76]. They found alcohol-attributable mortality to be strongly distributed to the disadvantage of persons with a low SES. 

Other studies assessed intersectional factors related to alcohol use in pregnancy. For example, analyses of the social determinants of health underpinning foetal alcohol spectrum disorder (FASD) in South Africa add critical insight from an intersectional feminist perspective [77]. The authors used an intersectionality wheel to conceptualize how the social and structural determinants of FASD identified in the literature are interconnected and indicative of broader inequalities shaping the lives of the affected women and children. Key intersecting social determinants that facilitate drinking during pregnancy among marginalized populations in South Africa include social norms and knowledge around drinking, drinking during pregnancy, alcohol addiction, and biological dependence, gender-based violence, inadequate access to contraception and abortion services, trauma and mental health concerns, moralization, and stigma. 

Another systematic review identified demographic, health and psychosocial variables associated with alcohol consumption during pregnancy which may lead to FASD [78] and identified the significance of prior mental illness, anxiety, depression, exposure to abuse and/or domestic violence, and the alcohol consumption behaviours of partners and family members as strong predictors of risky alcohol consumption during pregnancy and associated risk of FASD. 

Studies such as these indicate another range of complex interconnecting factors that need consideration when trying to reach pregnant women with respect to low-risk drinking guidelines. However, there was little representation of pregnant women’s experiences and perspectives in the studies reviewed, and limited analyses of how these determinants intersect with one another and relate to the broader structural factors to influence pregnancy outcomes.

## 5. Discussion

Overall, research on sex- and gender-related factors affecting substance use is lagging [12,79] due to oversights or omissions in research design, clinical trials participation, conflations of sex and gender concepts, and lack of integration of sex and gender measures and outcomes. This lag has particularly affected our knowledge base on women’s use of alcohol and the impacts of others’ alcohol use on women. 

However, evidence suggests that a range of sex-related factors affecting the ingestion of alcohol matter in determining the impact on women and specific impacts on female bodies [15]. In short, females are more damaged by alcohol on lesser amounts of alcohol related to sex-related factors affecting the anatomy, absorption, body fat/water ratio, and differing enzyme activity when compared to males. In addition, alcohol use is linked to numerous disease conditions, such as cancer. The impact of alcohol on breast cancer is of particular importance to females and women [15,16]. Thus, messages conveyed through low-risk drinking guidelines need to clearly convey how sex-related factors matter to bodily impact and disease processes.

Gender also matters to understanding the impacts of alcohol use by women and by others, which may affect women in real world situations. Relationship dynamics, drinking patterns and social expectations regarding alcohol use can have an impact on harms to women, such as binge drinking, IPV and sexual assault [25,80]. Thus, messaging in low-risk drinking guidelines needs to identify these and other gender-related vulnerabilities related to alcohol use. 

Sex and gender interact to produce singular patterns of impacts on women, such as alcohol use in response to childhood trauma [56], during social settings or in the context of pregnancy and mothering [59]. Such situations are unique to women and girls and reinforce a range of biological and social factors that operate together in the context of myriad intersectional factors, characteristics, and processes. Factors such as poverty and age, operating in the context of wider processes, such as sexism, racism, and colonialism, further delineate and construct the lived experiences of women.

We found that much of the research on alcohol and sex is based on a ‘sex differences’ paradigm [81]. While important in signalling differential male/female impacts and pointing to needed research on factors and processes, it is conceptually limiting, precluding a focus on sex-(or gender) related factors [81]. The research on gender and alcohol is inherently reflective of the cultural composition of populations studied and reflects temporal understandings of gender norms and attitudes. 

In the countries mentioned in the Introduction, there are varying degrees of cultural diversity, underscoring the importance of a gendered intersectional lens in building and assessing evidence and designing low-risk drinking guidelines that reflect the impacts of culture, SES, education, ethnicity, sexual orientation, and age. Advances in knowledge translation and implementation suggest that it is important to work with subpopulations to tailor messaging to effectively convey sex/gender and equity-related vulnerabilities related to alcohol use beyond basic population level messaging [82]. 

Despite different population and diversity profiles, many high-income countries have access to the same evidence and may experience similar trends in sex- and gender-related impacts. However, they do not uniformly translate the sex- and gender-related evidence into health promotion oriented low-risk drinking guidelines. We maintain that the evidence, while incomplete, is indicative of sex-specific factors that bear mentioning in health promotion, clinical guidance, and public education. Conveying sex-related differences is especially important for individuals in assessing their own risk and potentially changing their behaviours. 

The evidence on gender-related factors is less easy to translate in widely diverse sub-cultural groups in various countries. However, there may be broad universals based on gender that reflect both dominant and culturally specific masculinities and femininities that produce different social and interpersonal responses to girls, women, boys, and men and gender and sexual minorities who may be using alcohol, intoxicated, vulnerable, incapacitated, binge drinking, or occasionally drinking.

This synthesis of the literature has certain limitations. We did not conduct a quality assessment of the included studies as the main focus of our review was to build upon the emerging evidence on how alcohol affects women’s health in order to address sex- and gender-related factors in low-risk drinking guidelines. The studies synthesized in this review were conducted in high-income countries, such as the United States, the United Kingdom, and some countries from the European Union. 

These results may not be wholly applicable to other countries with different approaches to gender issues, women’s health or the inclusion of sex and gender in policy. There is confusion and conflation in the language used in the included papers, which can make interpretation difficult. For example, although we separate sex (biological) and gender (sociocultural)-related factors, the language that some of the studies used is imprecise and, in some cases, sex and gender were used interchangeably, causing us to interpret the findings. Finally, there are no standard definitions regarding what a standard drink is or what a social drinker is, and the findings from one study cannot be generalized to another study.

## 6. Conclusions

Both sex and gender impact alcohol use in a range of different ways. This is generally more damaging to the health and well-being of females; however, all genders experience differential social and biological factors that impact their health. Sex-related findings have implications for impaired function, inhibition, impulsivity, subjective experiences, and behavioural impacts. Gender-related findings have implications for responses to marketing, susceptibility to sexual assault or other harms, and stigma, stereotyped responses, and policies. Clearly, these types of evidence can be meaningfully reflected in low-risk drinking messages and guidance.

Messages in support of sex-specific lower risk drinking guidelines can include information on the faster and more damaging effects of alcohol for women, thus helping women gauge how much and how frequently they will drink. Information on bodily damage from alcohol, such as liver damage and injury, is important to know, especially when it occurs at comparably lower amounts of alcohol consumption. Finally, of particular interest to women is that alcohol use increases the risk of breast cancer, which could affect personal decision-making regarding alcohol. 

Other messages reflecting sex/gender interactions could cover the increased risks of faster intoxication among girls and women as well as how to monitor and resist social pressures that encourage binge drinking. Important gendered links between alcohol use and risks of intimate partner violence and sexual assault, in couple, dating or stranger situations whether used by perpetrators, victims, or both, need to be highlighted to heighten the awareness of collateral risk and to assist in reducing victim-blaming. Messages aimed at men and boys to encourage attitudes and behaviours that prevent the exploitation of intoxicated girls and women are potentially useful in this realm, by highlighting gendered impacts that negatively affect women and girls.

Translating this information into guidelines and other health promotion is clearly a choice for nations and/or the institutions responsible for public health. Some choose not to include this sex- and gender-specific evidence, perhaps based on overarching risk curves that imply little population-based sex difference in overall morbidity and mortality from alcohol [6,9,83]. While such trends could be due to differential amounts ingested by men and women per sitting and per year, they may obscure the immediate and long-term health impacts for individual women and men. Hence, inclusion and publication of sex- and gender-specific guidance is a way to reduce the overall risk by improving the overall health knowledge and engaging individual citizens in self-assessment and monitoring their risk from alcohol use. Such goals are in keeping with contemporary approaches to health promotion, health literacy, and precision healthcare.

## Data Availability

Not applicable.

## Supplementary Materials

The following supporting information can be downloaded at: https://www.mdpi.com/article/10.3390/ijerph19084523/s1, Table S1: Search Strategy.
