# Supplementary material for: Sex, Gender, and Alcohol Use: Implications for Women and Low-Risk Drinking Guidelines
Source: Int J Environ Res Public Health. 2022 Apr 8;19(8):4523. doi: 10.3390/ijerph19084523 (PMC9028341; doi:10.3390/ijerph19084523)
Supplement: Supplementary file 1 [file ijerph-19-04523-s001.zip › ijerph-1631776-supplementary.pdf]

Table S1. Search terms for search strategy conducted by an information specialist at the CCSA

("gender related" or "gender difference*" or "gender disparit*").ti,ab.
("sex related" or "sex difference*" or "sex disparit*").ti,ab.
"gender comparison*".ti,ab.
"sex comparison*".ti,ab.
"gender analys*".ti,ab.
"sex analys*".ti,ab.
tablle(transgender* or "trans gender*"). ti,ab.
("transsexual*" or "trans sexual*").ti,ab.
("non binar*" or nonbinar*).ti,ab.
exp Alcohol-Related Disorders/
exp Alcohol Drinking/
(binge drink* or underage drink* or under-age drink* or problem drink* or heavy drink* or harmful drink* or alcoholi* or inebriat* or intoxicat*).ti,ab.
("alcohol dependen*" or "alcohol misuse*" or "alcohol mis-use*" or "alcohol abuse*" or "alcohol overuse*" or "alcohol over-use*" or "alcohol addict*").ti,ab.
alcohol.ti,ab.
Alcohol Abstinence/
exp Risk Reduction Behavior/
("risk reduction" or "reducing risk" or "reducing risks" or "risk minimization" or "minimizing risk" or "minimizing risks" or "risk minimisation" or "minimising risk" or "minimising risks").ti,ab.
